# Microbial and clinical disparities in pneumonia: insights from metagenomic next-generation sequencing in patients with community-acquired and severe pneumonia

**DOI:** 10.3389/fmicb.2025.1538109

**Published:** 2025-06-20

**Authors:** Wang Luo, Shuhua Zhang, Jinhuan Sun, Jianhui Xu, Weihua Huang, Ruiqing Hao, Zhao Ou, Ziyang Wen, Daiwei Wang, Guanhua Xiao, Hangming Dong

**Affiliations:** ^1^Department of Respiratory and Critical Care Medicine, The Zengcheng Branch of Nanfang Hospital, Southern Medical University, Guangzhou, China; ^2^Dian Diagnostics Group Co. Ltd., Hangzhou, China; ^3^Department of Medical, Guangzhou Dian Diagnostics Group Co. Ltd., Guangzhou, China; ^4^Department of Respiratory and Critical Care Medicine, Nanfang Hospital, Southern Medical University, Guangzhou, China

**Keywords:** community-acquired pneumonia, severe pneumonia, clinical characteristics, metagenomic next-generation sequencing, microbiological profiles

## Abstract

**Background:**

Community-acquired pneumonia (CAP) is a major global cause of death, with its varying symptoms and severity complicating diagnosis and treatment. Severe pneumonia (SP), a more critical form of CAP, has higher mortality and often requires intensive care. The identification of clinical markers to differentiate CAP from SP has the potential to improve treatment protocols and patient outcomes. Concurrently, metagenomic next-generation sequencing (mNGS) demonstrates significant promise in pathogen detection and in elucidating microbiome disparities between CAP and SP.

**Methods:**

This retrospective study analyzed clinical and pathogen data from 204 patients diagnosed with CAP and 25 patients diagnosed with SP in the Department of Respiratory and Critical Care Medicine at the Zengcheng Branch of Nanfang Hospital, Southern Medical University, spanning the period from September 2022 to June 2023. Clinical characteristics were compared, and bronchoalveolar lavage fluid (BALF) samples underwent mNGS for microbial detection and characterization. Statistical analyses, encompassing Chi-square, Fisher’s exact test, Student’s *t*-test, and LEfSe analysis, were employed to compare clinical and microbiological data between the CAP and SP cohorts.

**Results:**

Patients with SP were significantly older and exhibited higher incidences of sepsis, hypotension, tachycardia, multilobar infiltrates, and consciousness disorders compared to those with CAP. Elevated levels of C-reactive protein (CRP) and procalcitonin (PCT) were more frequently observed in SP patients. mNGS analysis identified diagnostic microbiology profiles between groups. Diverse microbiological profiles (> 5 species) were more common in SP patients (> 30% detection rate). Beta diversity analysis demonstrated significant differences in microbial community composition between CAP and SP groups (*p* = 0.001), though alpha diversity metrics showed no significant differences. Both LEfSe and ANCOM-BC2 analyses consistently identified *Pseudomonas* as a potential biomarker for SP and *Streptococcus* for CAP.

**Conclusion:**

The substantial differences observed in clinical characteristics, pathogen profiles, and microbiomes between patients with CAP and those with SP highlight the imperative need for comprehensive diagnostic methodologies in the management of pneumonia. mNGS has demonstrated substantial utility in informing personalized treatment strategies, with the potential to enhance clinical outcomes. Future research should prioritize elucidating the dynamics of microbial communities and their impact on pneumonia severity, with the objective of refining and optimizing therapeutic strategies.

## Introduction

Pneumonia and other lower respiratory infections represent the fourth leading cause of mortality, making them the most lethal category of communicable diseases. CAP constitutes a major contributor to mortality rates among both adults and children worldwide. The overall mortality rate of CAP typically range from 5% to 10%, with 10 to 15 million hospitalizations every year (Wang et al., 2023). The variability in clinical presentations, causative microorganisms, and severity indicators renders the accurate diagnosis and management of CAP exceptionally challenging (Lanks et al., 2019). A delay in diagnosing and treating CAP can have serious consequences. This progression can exacerbate the condition into SP, thereby markedly impacting patient treatment outcomes and prognosis (Wang et al., 2023). Studies have shown that 2% to 24% of SP patients require admission to an ICU (Marti et al., 2012; Wang C. et al., 2024) and risk of hospital mortality significantly increased (Johnstone et al., 2023). Hence, it is important for physicians to be able to distinguish between CAP and SP patients and to administer timely treatment to prevent the pneumonia from deteriorating.

The primary etiology of pneumonia is the infection by pathogens, with bacteria playing a predominant role in adult pneumonia cases. Additionally, there is substantial evidence indicating the common occurrence of viral-bacterial co-infections (Liu Y. et al., 2023). So timely identification of causative agents and investigation of differences in microorganism between CAP and SP patients are significant for effective treatment and management of pneumonia (Wang C. et al., 2024). Traditional pathogens diagnostic methods including tissue biopsy, immunology, microscopy, time of flight mass spectrometers, culture, Xpert MTB/RIF Ultra, polymerase chain reaction (PCR) show various diagnostic ability, however, they all exhibit limitations in diagnosing mixed infections, sensitivity, processing time, and the range of detectable pathogens (Liu Z. et al., 2023; Zhang et al., 2020). As next-generation sequencing has developed rapidly during the past decade, mNGS has become an important tool in precision medicine due to high sensitivity, high timeliness, easy sample processing, and a wide range of detectable panels. To date, numerous studies have examined the value of mNGS in identifying pathogens and reducing the duration of empirical treatment in the absence of pathogenic evidence (Wilson et al., 2019; Yang et al., 2022; Zhu et al., 2023). In previous studies, the pulmonary microbiota, immune system activity, and pathogen aggressiveness have been primarily examined as factors contributing to SP in patients with CAP. In contrast to the balanced lung microbial community, characterized by a small yet highly diverse bacterial population, patients with severe pneumonia exhibit a dysregulated lung microbiome (Wang et al., 2020; Wang B. et al., 2024). Clinically, given the elevated risk of clinical deterioration in SP patients, pulmonologists frequently resort to broad-spectrum antibiotic therapy. However, the diminished immune function and microbial resistance observed in patients with SP significantly limit the efficacy of antibiotics in inhibiting pathogens. Furthermore, it is important to highlight the absence of detailed comparative analyses of the lower respiratory tract microbiota between patients diagnosed with CAP and those with SP.

This study seeks to elucidate potential differences in the microbiota distribution within the lower respiratory tract between patients with CAP and those with SP by utilizing mNGS. Using this advanced approach, it is possible to identify bacteria, eukaryotes, and viruses unbiasedly at the species level (Chen et al., 2021). We compared the differences of clinical characteristics between CAP and SP patients, then analyzed the microorganism findings by mNGS in two groups after evaluating detection performance of mNGS to culture. Aiming to explore special clinical features and pathogens markers that can distinguish CAP and SP, as well as indicate the aggravation of pneumonia.

## Materials and methods

### Patients and definitions

We retrospectively studies 243 pneumonia patients from September 2022 to June 2023 in the Department of Respiratory and Critical Care Medicine at the Zengcheng Branch of Nanfang Hospital. Excluding patients with incomplete data collection, we initially screened 204 CAP and 25 SP patients. CAP was diagnosed in patients presenting with acute lower respiratory tract symptoms (cough, sputum production, dyspnea, and fever ≥ 38°C), laboratory evidence of systemic inflammation (C-reactive protein [CRP] ≥ 30 mg/L, procalcitonin [PCT] ≥ 0.25 ng/mL, or leukocyte count > 10 × 10^9^/L or < 4 × 10^9^/L), and new pulmonary infiltrates on chest radiography or CT, after excluding non-infectious etiologies (e.g., pulmonary edema, malignancy), in alignment with IDSA/ATS guidelines (Metlay et al., 2019). SP was defined as meeting either one major criterion (mechanical ventilation via tracheal intubation or septic shock requiring vasopressor therapy despite adequate fluid resuscitation) or ≥ 3 minor criteria (respiratory rate ≥ 30 breaths/min, oxygenation index ≤ 250 mmHg, multilobar infiltrates, altered mental status, blood urea nitrogen ≥ 7.14 mmol/L, or systolic blood pressure < 90 mmHg requiring fluid resuscitation). Disease severity was further stratified using the Pneumonia Severity Index (PSI) and Quick Sepsis-related Organ Failure Assessment (qSOFA) scores. Patients fulfilling SP criteria were enrolled, and full clinical characteristics, including symptom profiles and inflammatory markers, are detailed in Supplementary Table 2 (Cao et al., 2018).

### Data collection

The data were collected retrospectively from medical records (including patients backgrounds, systemic underlying disease, severity score, signs and symptoms of clinical presentation, laboratory findings, outcome of clinical and molecular diagnosis results of pathogens), but we did not obtain any follow-up results. The reported data are all a result of routine clinical activities.

### mNGS sequencing

BALF samples were collected from patients within 48 h of hospital admission. 1 mL of samples was centrifuged to collect the pathogens and human cells at 12,000 × *g* for 5 min. 50 μL of precipitate was treated with 0.5% Tween 20 (Sigma) and 1 U of Benzonase (Sigma), followed by incubation at 37°C for 5 min to deplete host nucleic acids. After transferring the product to fresh tubes, 500 μL of ceramic beads were added to each, followed by bead beating using a Minilys personal TGrinder H24 homogenizer (Tiangen). Purified nucleic acid, extracted using a QIAamp UCP pathogen minikit (Qiagen), was eluted in 60 μL of elution buffer. Nextera DNA Flex kit (Illumina) was carried out according to manufacturer’s protocol for DNA libraries construction, followed by purification and size selection. Library quality was evaluated by an Agilent 2100 Bioanalyzer (Agilent Technologies). Subsequently, the libraries were normalized, pooled (1.5 pM) and sequenced on an Illumina NextSeq 550 sequencer. Metagenomic next-generation sequencing (mNGS) of BALF samples was completed in 48 h. The raw sequence data reported in this paper have been deposited in the Genome Sequence Archive (Genomics, Proteomics & Bioinformatics 2021) in National Genomics Data Center (Nucleic Acids Res 2022), China National Center for Bioinformation/Beijing Institute of Genomics, Chinese Academy of Sciences (GSA: CRA024916) that are publicly accessible at https://ngdc.cncb.ac.cn/gsa.

### mNGS data analysis

Trimmomatic was used to remove undesired reads such as reads with adapter contamination, duplicate reads, low-quality reads, and reads shorter than 70 bp (Bolger et al., 2014). As for the reads with low-complexity, they were removed using Komplexity (parameters by default). To construct the microbial genome database, it was necessary to exclude human sequences. Therefore, reads were first mapped to hg38 human genome database employing SNAP v1.0beta.18. Thereafter, the selection of pathogens and their corresponding genomes or assemblies was conducted according to the Kraken2 criteria, which are designed to select representative assemblies for microorganisms (viruses, bacteria, protozoa, fungi, and other multicellular eukaryotic pathogens) from the NCBI Genome and Assembly databases.^1^ Microbial reads were mapped to the database using BWA software (Li and Durbin, 2009). Reads that had more than 90% of their bases mapped were defined as mapped reads. In the subsequent analysis, only reads mapped to the genome of same species were included and reads with multiple locus alignments within the same genus were excluded. Results were deemed positive for bacteria (excluding mycobacteria) and viruses when their sequence count was 10 times higher than other microorganisms. Fungi were positive if their sequence count was 5 times higher than other fungi. Mycobacterium tuberculosis was considered positive with at least one species or genus-level read matching the reference genome. Additionally, a pathogen was positive if conventional culture detected it and mNGS reads exceeded 50.

### Statistical analysis

For the clinical characteristics of patients (Table 1), the mNGS detection rate (Supplementary Table 1), and the relative abundance of non-causative respiratory microflora (Supplementary Table 2), using the Chi-squared test or Fisher’s exact test, categorical variables were analyzed, and continuous variables were analyzed using the Student’s *t*-test or the non-parametric Mann-Whitney U test. We shown median (IQR) for continuous variables with non-normal distribution (C-reactive protein level, procalcitonin level, hematocrit, white blood cells, platelets, lymphocytes, neutrophils and relative abundance of non-causative respiratory microflora) and mean ± SD for those with normal distribution (age[years] and length of hospital stay). Statistical analyses were conducted using GraphPad Prism 12 software.

**TABLE 1:** CAP and SP patients’ baseline characteristics.

	CAP (%)	SP (%)	*P*-value^a^
Patient count	204	25	
**Background**			
Age (years), mean ± standard deviation	50.7 ± 16.4	69.7 ± 16.4	< 0.0001
Age ≥ 65 years	48 (23.5)	18 (72.0)	< 0.0001
Gender (M/N)	111/93	23/2	0.0002
**Systemic underlying disease**			
Cancer	191 (93.6)	22 (88.0)	0.3940
Congestive heart failure	194 (95.1)	16 (64.0)	< 0.0001
Cerebrovascular disease	185 (90.7)	15 (60.0)	0.0002
Chronic kidney disease	175 (85.8)	22 (88.0)	1.0000
Chronic liver disease	140 (68.6)	17 (68.0)	1.0000
Type 2 diabetes mellitus	22 (10.8)	4 (16.0)	0.4998
Sepsis	0	2 (8.0)	0.0115
**PSI stage**			
I	41 (20.1)	0	0.0103
II	91 (44.6)	2 (8.0)	0.0003
III	39 (19.1)	2 (8.0)	0.2674
IV	30 (14.7)	8 (32.0)	0.0426
V	3 (1.5)	13 (52.0)	< 0.0001
**Vital signs**			
Systolic blood pressure < 90 mmHg	1 (0.5)	12 (48.0)	< 0.0001
Temperature < 35C° or >40C°	1 (0.5)	1 (4.0)	0.2068
Pulse ≥ 125 bpm	7 (3.4)	8 (32.0)	< 0.0001
Multilobar infiltrates	134 (65.7)	24 (96.0)	0.0010
Consciousness disorders	2 (1.0)	13 (52.0)	< 0.0001
**Initial treatment**			
Mechanical ventilation	3 (1.5)	16 (64.0)	< 0.0001
Vasodilators	3 (1.5)	13 (52.0)	< 0.0001
Fluid resuscitation	1 (0.5)	12 (48.0)	< 0.0001
**Laboratory findings**			
C-reactive protein level (mg/L), median (IQR)	23.0 (5.4–66.2)	70.6 (50.5–161.8)	< 0.0001
C-reactive protein level ≥ 100 mg/L	27 (13.2)	11 (44.0)	0.0005
Procalcitonin level (ng/mL), median (IQR)	0.07 (0.04–0.14)	0.39 (0.19–2.64)	< 0.0001
Procalcitonin level ≥ 2 ng/mL	7 (3.4)	9 (36.0)	< 0.0001
HCT (%), median (IQR)	38.8 (36.1–42.1)	34.1 (30.8–37.3)	< 0.0001
WBC count (× 10^9^ cell/L), median (IQR)	7.1 (5.6–9.3)	11.2 (7.6–15.2)	0.0002
PLT count (× 10^9^ cell/L), median (IQR)	230.5 (185.0–289.0)	268.0 (209.0–300.0)	0.2782
LYM count (× 10^9^ cell/L), median (IQR)	1.5 (1.1–1.9)	0.9 (0.6–1.2)	< 0.0001
NEU count (× 10^9^ cell/L), median (IQR)	4.9 (3.3–7.0)	10.3 (6.4–12.5)	< 0.0001
**Outcome**			
Length of hospital stay, mean ± SD	8.4 ± 3.5	23.1 ± 16.8	< 0.0001

PSI, pneumonia severity index; bpm, beats per minute; HCT, hematocrit; WBC, white blood cells; PLT, platelets; LYM, lymphocytes; NEU, neutrophils.

^*a*^Statistical difference between CAP and SP were evaluated by Chi-square Test or Fisher’s exact test for categorical variables and the Mann–Whitney U test for continuous variables.

The species count and relative abundance tables for each site were input into Python for statistical analysis. The alpha diversity of the microbiota profile for each subject was evaluated by group at different data levels using the scikit-bio package (version 0.6.2) in Python. With the scikit-bio package (version 0.6.2) in Python, multivariate permutational analysis of variance (PERMANOVA) and principal component analysis (PCA) were performed for intergroup comparisons. The results of the Principal Coordinates Analysis (PCoA) were obtained using the scikit-bio package (version 0.6.2) in Python. Associations between specific microbial species or genera and patient parameters were identified through the Linear Discriminant Analysis Effect Size (LEfSe) method (Chen et al., 2022). The LDA score greater than 2 was considered indicative of significant differences.

### Community structure difference test

To evaluate the potential association between inflammatory marker levels and microbial community composition, we performed a Mantel test. This test was used to assess the correlation between inflammatory differences (CRP/PCT levels) and the microbial distance matrix, which was calculated based on Bray–Curtis dissimilarity. Statistical significance was determined using 9,999 permutations, and the analysis was conducted in R using the vegan package. The Bray–Curtis dissimilarity index was calculated to assess the differences in microbial community structure. PERMANOVA (permutational multivariate analysis of variance) was performed with 999 permutations to assess the joint effects of clinical variables, including CRP, PCT, and age, on the variation in respiratory microbiome structure. To evaluate spatial correlation, a Mantel test was performed to assess the correlation between inflammatory differences and the microbial distance matrix.

## Results

### Clinical characteristics of CAP and SP patients

During the study, 243 pneumonia patient specimens underwent metagenomics next-generation sequencing (mNGS), with 218 diagnosed with CAP and 25 with SP. After excluding incomplete data, 204 CAP and 25 SP cases were analyzed. Table 1 shows that SP patients were significantly older than CAP patients (mean age 69.7 vs. 50.7 years; *p* < 0.0001), with a higher proportion aged 65 or older (72.0% vs. 23.5%; *p* < 0.0001). CAP patients more often had cardiac insufficiency (*p* < 0.0001) and vascular disease of the brain (*p* = 0.0002), while sepsis was more frequent in SP patients (8.0% vs. 0%; *p* = 0.0115). Other comorbidities were similar between the groups. SP patients were more often in higher PSI stages IV and V (*p* = 0.0426 and *p* < 0.0001). They also had higher rates of systolic blood pressure < 90 mmHg, pulse ≥ 125 bpm, multilobar infiltrates, and consciousness disorders (all *p* < 0.0001, except *p* = 0.0010 for infiltrates) compared to CAP patients. Laboratory results revealed significantly higher median levels of CRP and PCT in SP patients (both *p* < 0.0001). Elevated CRP (≥ 100 mg/L) and PCT (≥ 2 ng/mL) were more common in SP patients (44.0% vs. 13.2%, *p* = 0.0005 and 36.0% vs. 3.4%, *p* < 0.0001), highlighting these biomarkers’ importance in differentiating CAP from SP. Other parameters such as hemoglobin, white blood cells, lymphocytes, and neutrophils, are also important, eflecting the increased severity of SP. SP patients had significantly longer hospital stays (*p* < 0.0001).

### Pathogenic microbiological findings between CAP and SP patients

**FIGURE 1:**
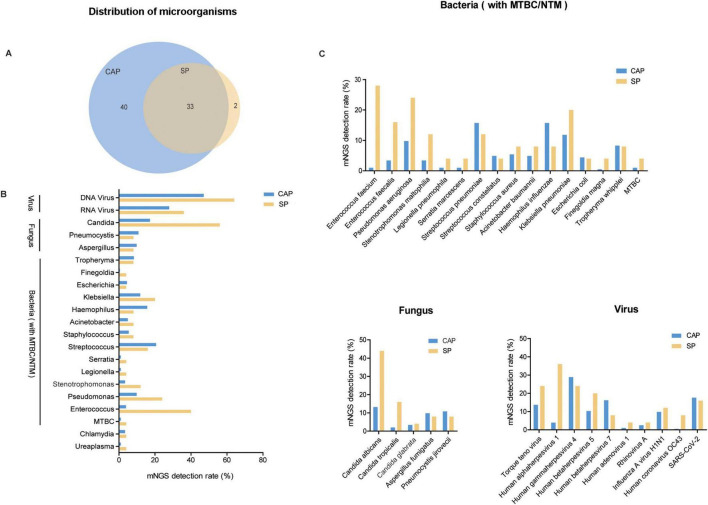
Identified microorganisms distribution in CAP and SP Groups. **(A)** Venn diagram showing the overlap of microbial species detected in CAP and SP groups. The numbers represent the unique and common microorganisms identified in each group. **(B)** Bar chart displaying the detection rate (%) of various microorganisms across different categories (viruses, fungi, and bacteria) in CAP (blue) and SP (yellow) groups. The categories encompass DNA viruses, RNA viruses, *Candida*, and various bacterial species, highlighting distinct differences in microbial distribution patterns between the two groups. **(C)** Comprehensive analysis of detection rates (%) for bacterial, fungal, and viral species in CAP and SP groups, with detailed species-level characterization. The chart demonstrates differential microbial compositions across pneumonia types.

This study analyzes the diagnostic microbiology results obtained through mNGS. According to Figure 1A, mNGS detected 73 distinct microorganisms in the CAP group and 35 in the SP group, with 40 unique to CAP and 2 to SP. Due to detection rates below 5% for these unique organisms, the analysis concentrated on the 33 commonly detected microorganisms. Bacterial species constituted the majority of identified organisms in both groups (Figures 1B, C). In CAP patients, the leading pathogens were *Streptococcus pneumoniae*, *Haemophilus influenzae*, and *Klebsiella pneumoniae*, while in SP patients, they were Enterococcus faecium, *Pseudomonas aeruginosa*, and *Klebsiella pneumoniae*. The detection rate of *Klebsiella pneumoniae* using mNGS was significantly higher in cases of SP compared to CAP. SP patients also showed higher detection rates of Enterococcus faecalis, *Pseudomonas aeruginosa*, and *Stenotrophomonas* maltophilia compared to CAP patients (Figure 1C). Fungal pathogens, especially *Candida albicans* and *Candida tropicalis*, were more common in SP patients, indicating they might mark pneumonia severity. SP patients also had higher rates of *Human alphaherpesvirus 1* (HSV1), whereas CAP patients had more Human gammaherpesvirus 4 (EBV) and Human coronavirus.

### Detection patterns of multiple microorganisms in CAP and SP patients

**FIGURE 2:**
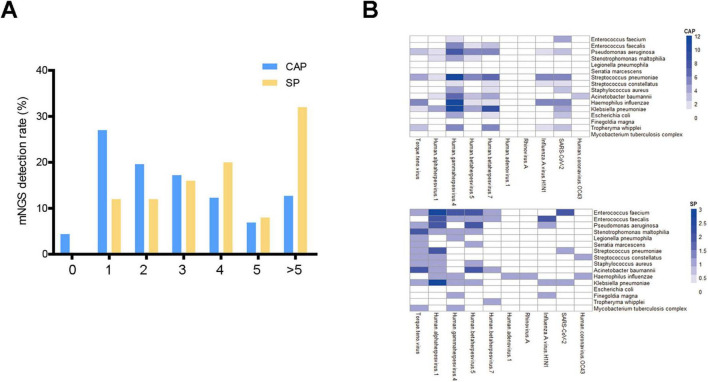
Multiple pathogen detection patterns in CAP and SP Groups. **(A)** Bar graph showing the number of microorganism detected in CAP and SP groups. The *x*-axis indicates the number of microorganism (from none to more than five), and the *y*-axis shows the mNGS detection rate (%). **(B)** Heatmaps representing bacterial-viral interaction profiles in CAP and SP groups. The visualization demonstrates the frequency and intensity of polymicrobial associations between bacterial and viral species. Color intensity correlates with the frequency of concurrent microbial detection.

Our findings showed that SP patients more frequently harbored multiple microorganisms, especially with over five distinct species, where mNGS detection rates surpassed 30%, unlike the lower rates in CAP patients (Figure 2A). Both groups showed minimal negative detection results, around 3%. Single and dual organism detection was more common in CAP patients, while three to five organisms were similar between CAP and SP patients. Regarding viral-bacterial co-detection patterns, EBV was most frequently identified alongside bacterial species in CAP patients (37.7%), whereas Torque teno virus and HSV1 showed higher co-detection rates with bacterial species in SP patients (52% and 68%, respectively) (Figure 2B).

### Comparison of microbiome diversity

**FIGURE 3:**
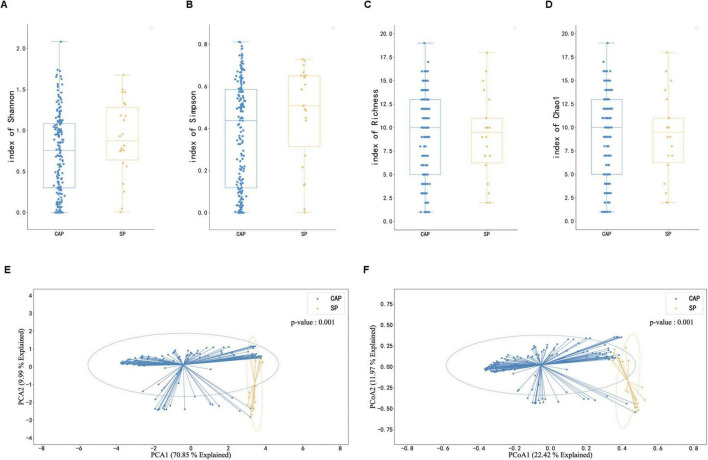
Comparison of microbiome diversity and composition between CAP and SP patients. **(A–D)** Box plots illustrating microbiome diversity metrics: Shannon index **(A)**, Simpson index **(B)**, Richness **(C)**, and Chao1 index **(D)** in CAP and SP groups. Higher values indicate greater diversity. **(E,F)** Principal Component Analysis (PCA) and Principal Coordinate Analysis (PCoA) plots of mNGS data from BALF samples. **(E)** PCA plot showing the first two principal components explaining 79.85% of the total variance. **(F)** PCoA plot with PCoA1 (explaining 22.42% of variance) on the *x*-axis and PCoA2 (explaining 13.97% of variance) on the *y*-axis. In both plots, blue dots represent CAP samples and orange dots represent SP samples. Ellipses indicate 95% confidence intervals.

The study conducted a comprehensive analysis of microbiome diversity between CAP and SP groups using multiple alpha diversity metrics and beta diversity analysis. For alpha diversity assessment, we evaluated Shannon index, Simpson index, Richness index, and Chao1 index (Figures 3A–D). The analysis revealed that while the SP group showed a trend toward higher median Shannon and Simpson indices compared to the CAP group, these differences did not reach statistical significance (*p* > 0.05). Conversely, the Richness and Chao1 indices displayed a trend toward higher values in the CAP group, though again without statistical significance. This complex pattern of alpha diversity metrics suggests that the relationship between disease type and microbiome diversity is nuanced and requires careful interpretation. Beta diversity analysis, performed using Principal Coordinates Analysis (PCoA), demonstrated significant differences in microbial community composition between CAP and SP groups (*p* = 0.001, Figures 3E, F). The clear separation of samples in the PCoA plot indicates distinct microbial community structures between these two pneumonia types, despite the subtle differences in alpha diversity metrics. The CAP group samples clustered more tightly, suggesting a more consistent microbial community structure, while SP group samples showed greater dispersion, potentially indicating more heterogeneous microbial compositions among severe pneumonia patients. These findings highlight that while overall species richness and evenness may not significantly differ between CAP and SP, the specific composition and structure of the microbial communities are distinctly different between these two clinical entities. This distinction in community composition, rather than simple diversity metrics, may be more relevant for understanding disease mechanisms and improving diagnostic approaches.

### Taxa differences between groups

**FIGURE 4:**
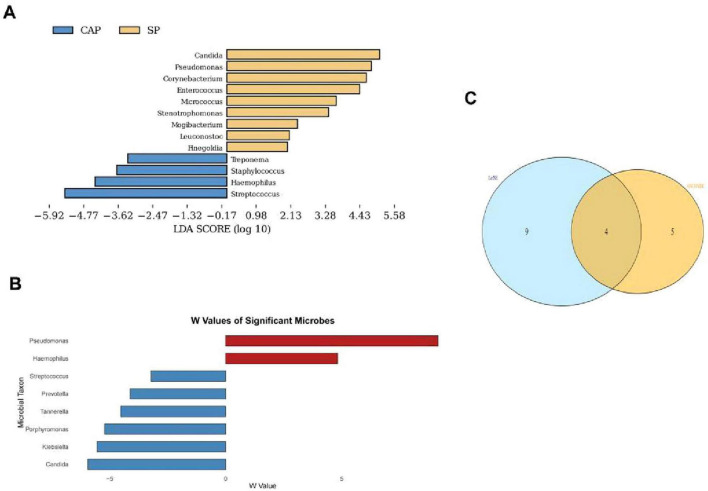
Microbial taxonomic disparities between CAP and SP groups identified by dual analytical approaches. **(A)** LEfSe-derived Linear Discriminant Analysis (LDA) scores (log10-transformed) showing significantly enriched taxa in CAP (blue bars) and SP (red bars). Threshold: LDA > 2.0. **(B)** ANCOM-BC2 analysis of microbial effect sizes (W values), with *Pseudomonas* (W = 6.0) and *Candida* (W = 5.2) demonstrating strongest SP associations. Negative W values indicate CAP enrichment. **(C)** Venn diagram quantifying methodological concordance: 4 overlapping taxa (*Candida*, *Pseudomonas*, *Haemophilus*, *Streptococcus*) were identified by both LEfSe (*n* = 9) and ANCOM-BC2 (*n* = 7).

Dual analytical approaches (LEfSe and ANCOM-BC2) were employed to identify microbial taxa differentially abundant between CAP and SP groups, with results visualized in Figure 4. LEfSe analysis (Figure 4A) revealed *Treponema* (LDA = 5.92), *Staphylococcus* (LDA = 4.73), and *Haemophilus* (LDA = 3.62) as CAP-enriched taxa, while *Candida* (LDA = 7.31), *Pseudomonas* (LDA = 6.84), and *Corynebacterium* (LDA = 4.02) were associated with the SP group. ANCOM-BC2 analysis (Figure 4B) identified different taxa associations. Comparing the two methods, only *Streptococcus* was consistently associated with CAP, while only *Pseudomonas* was consistently associated with SP across both analyses. This limited overlap highlights the complexity of microbial dynamics in pneumonia and the importance of using multiple analytical approaches. Venn diagram analysis (Figure 4C) illustrated the differences between the two methods, with several taxa uniquely identified by each approach. LEfSe uniquely identified *Micrococcus* and *Stenotrophomonas*, while ANCOM-BC2 solely identified *Prevotella* (W = −4.8) and *Klebsiella* (W = 3.5). These findings collectively highlight *Pseudomonas* as a potential biomarker for SP and *Streptococcus* for CAP, supported by both analytical methods. However, the discrepancies observed between LEfSe and ANCOM-BC2 results for other taxa underscore the need for cautious interpretation and further investigation to clarify the roles of these microbes in pneumonia severity.

## Discussion

Our study reveals the differences of key clinical biomarker between CAP and SP patients. The older age and higher number of elderly patients in the SP group highlight age as a crucial risk factor for severe disease. Previous studies (Wang et al., 2023; Wu et al., 2023; Yi et al., 2024) indicate that SP patients have significantly higher levels of C-reactive protein and procalcitonin compared to CAP patients, suggesting a more severe inflammatory response. As compared to the CAP group, the SP group had significantly higher levels of CRP (100 mg/L) and PCT (2 ng/mL) (44.0% vs. 13.2%, *p* = 0.0005; 36.0% vs. 3.4%, *p* < 0.0001). This indicates that high CRP and PCT levels could be key markers for differentiating between CAP and SP. Furthermore, we investigated the relationship between inflammatory marker levels (CRP/PCT) and microbial community composition in patients with CAP and SP. To assess this association, we employed a Mantel test to examine the correlation between inflammatory differences and the microbial distance matrix based on Bray–Curtis dissimilarity (Supplementary Figure 1). Our findings indicated that there was no significant correlation between CRP/PCT levels and microbial community dissimilarity, suggesting that these inflammatory markers may not directly shape the overall microbial structure.

We then examined mNGS use in CAP and SP patients. Traditional pathogen detection methods, like culture, are limited by broad-spectrum antibiotics and slow-growing organisms. mNGS addresses these limitations and surpasses PCR by directly sequencing DNA and RNA from samples (Liu et al., 2022). The results of our study show a high sensitivity consistency between mNGS and culture, as indicated in Supplementary Table 1. mNGS results identified *Streptococcus pneumoniae*, *Haemophilus influenzae*, and *Klebsiella pneumoniae* as the top microorganisms in CAP patients. The significant presence of fungal species and herpesviruses in SP patients suggests possible secondary infections that may worsen the disease. The increased detection of *Candida species* and *Human alphaherpesvirus 1* in these patients underscores the necessity for thorough diagnostic methods to manage these co-infections (Lee et al., 2016).

The observed heterogeneity in alpha diversity trends between CAP and SP groups highlights the multifaceted nature of microbial community structure in different pneumonia etiologies. This complexity suggests that simple diversity metrics alone may not fully capture the relevant differences between these clinical entities. Instead, the significant distinctions observed in beta diversity analysis indicate that the specific composition and inter-relationships within the microbial communities, rather than overall diversity, may be more pertinent to understanding disease mechanisms and developing diagnostic approaches.

The partial inconsistency between LEfSe and ANCOM-BC analysis results in this study, particularly regarding the attribution of *Candida*, underscores the complexity of microbiome data analysis. These differences may arise from various factors, including but not limited to sample size, data distribution characteristics, and statistical assumptions of each analytical method. This reminds us of the need to consider results from multiple analytical approaches comprehensively when interpreting microbiome data, and to treat conclusions from a single analysis with caution. Future studies may require larger sample sizes and more diverse analytical strategies to address these inconsistencies. Nevertheless, the identification of *Pseudomonas* as a characteristic species of the SP group in both analyses strengthens its potential as a biomarker. This consistency also provides a solid foundation for further investigation into the role of *Pseudomonas* in the pathogenesis of severe pneumonia. These discrepancies indicate that patients suffering from severe pneumonia may possess a more intricate microbial ecosystem, potentially influencing both the severity of their condition and their responsiveness to therapeutic interventions (Cleary et al., 2015).

This study utilized mNGS technology to deeply analyze the lower airway microbiome, uncovering notable differences between CAP and SP patients. By examining BALF, we obtained key insights into the lower respiratory tract’s microbial environment. However, small sample size and single-center design of the study are limitations that future multi-center trials should address. Additionally, the retrospective analysis of BALF samples and potential dilution effects require careful consideration.

## Conclusion

In conclusion, this study elucidates significant differences in clinical characteristics, microbial compositions, polymicrobial patterns, and microbiome diversity between patients with CAP and those with SP, underscoring the critical importance of comprehensive diagnostic approaches in the management of pneumonia. Metagenomic next-generation sequencing has the potential to improve clinical outcomes when applied to guiding personalized treatment strategies. Future research should aim to further elucidate the mechanisms underlying microbial community dynamics and their impact on pneumonia severity, with the ultimate goal of enhancing treatment strategies for pneumonia.

## Data Availability

The dataset associated with this study is not publicly available due to confidentiality restrictions. However, the dataset can be made available upon reasonable request to the corresponding author, subject to approval and adherence to applicable regulations and conditions. Requests to access these datasets should be directed to HD, huxidhm@126.com.

## Footnotes

1
https://benlangmead.github.io/aws-indexes/k2
