# Supplementary figures and images for: Microbial and clinical disparities in pneumonia: insights from metagenomic next-generation sequencing in patients with community-acquired and severe pneumonia
Source: Front Microbiol. 2025 Jun 20;16:1538109. doi: 10.3389/fmicb.2025.1538109 (PMC12227010; doi:10.3389/fmicb.2025.1538109)

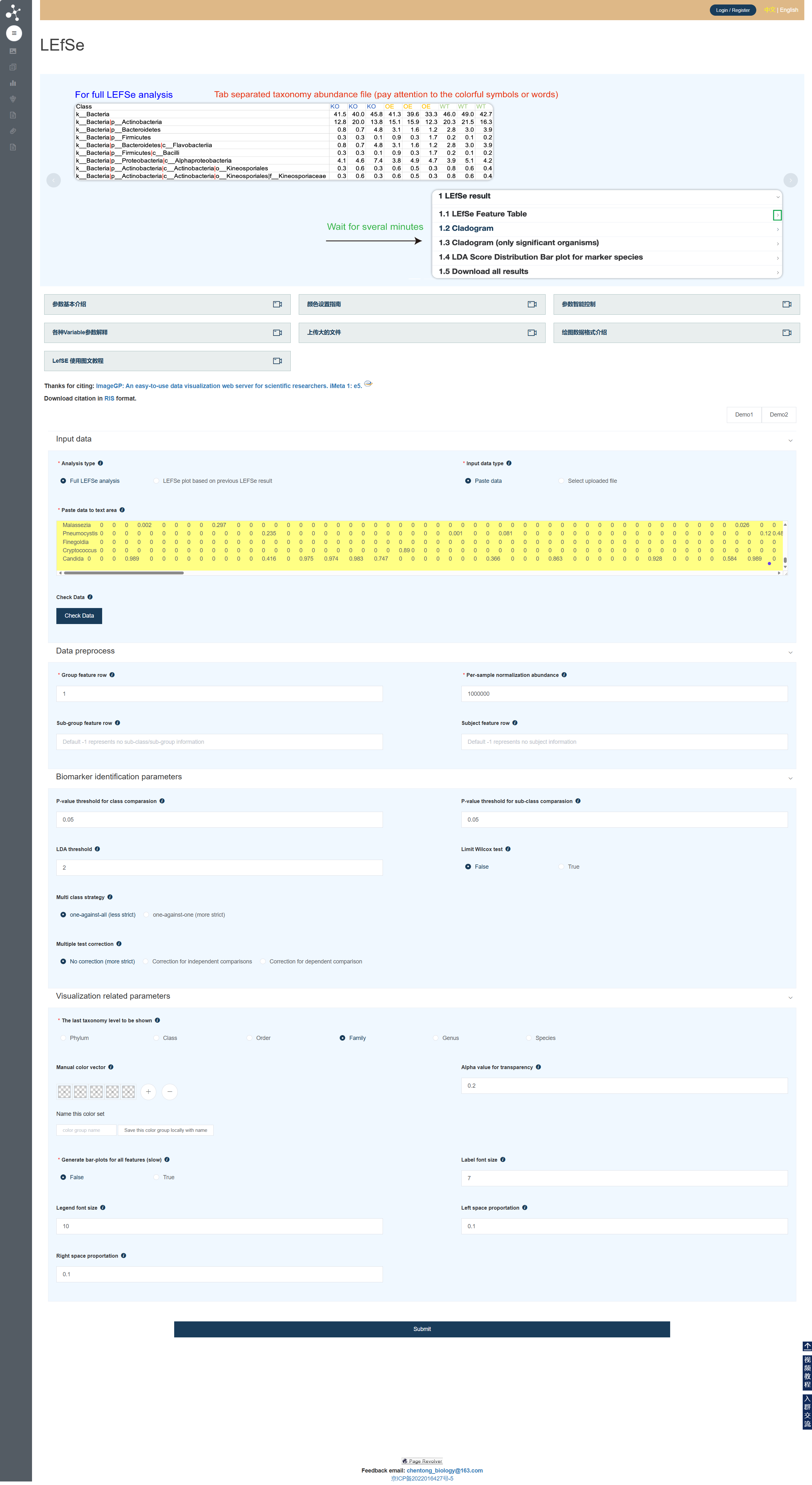

Supplement: Supplementary file 3 [file Data_Sheet_1.zip › Code Commit/Microecological Indicator Calculation/LEfSe_2024-07-30_170213.png]

W Values of Significant Microbes

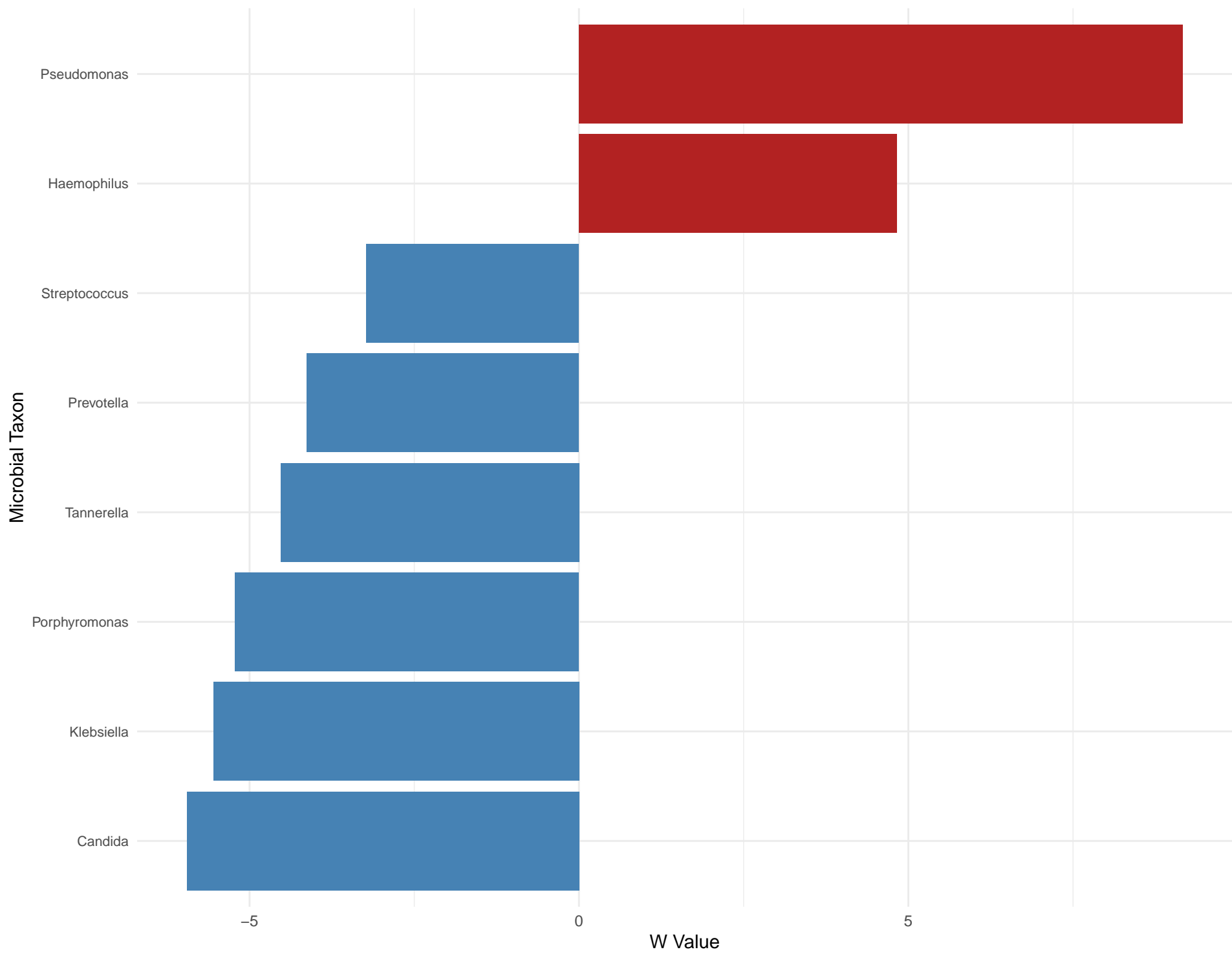

Supplement: Supplementary file 5 [file Data_Sheet_3.zip › ANCOMBC/ANCOMBC_significant_heatmap.pdf]

W Values of Significant Microbes

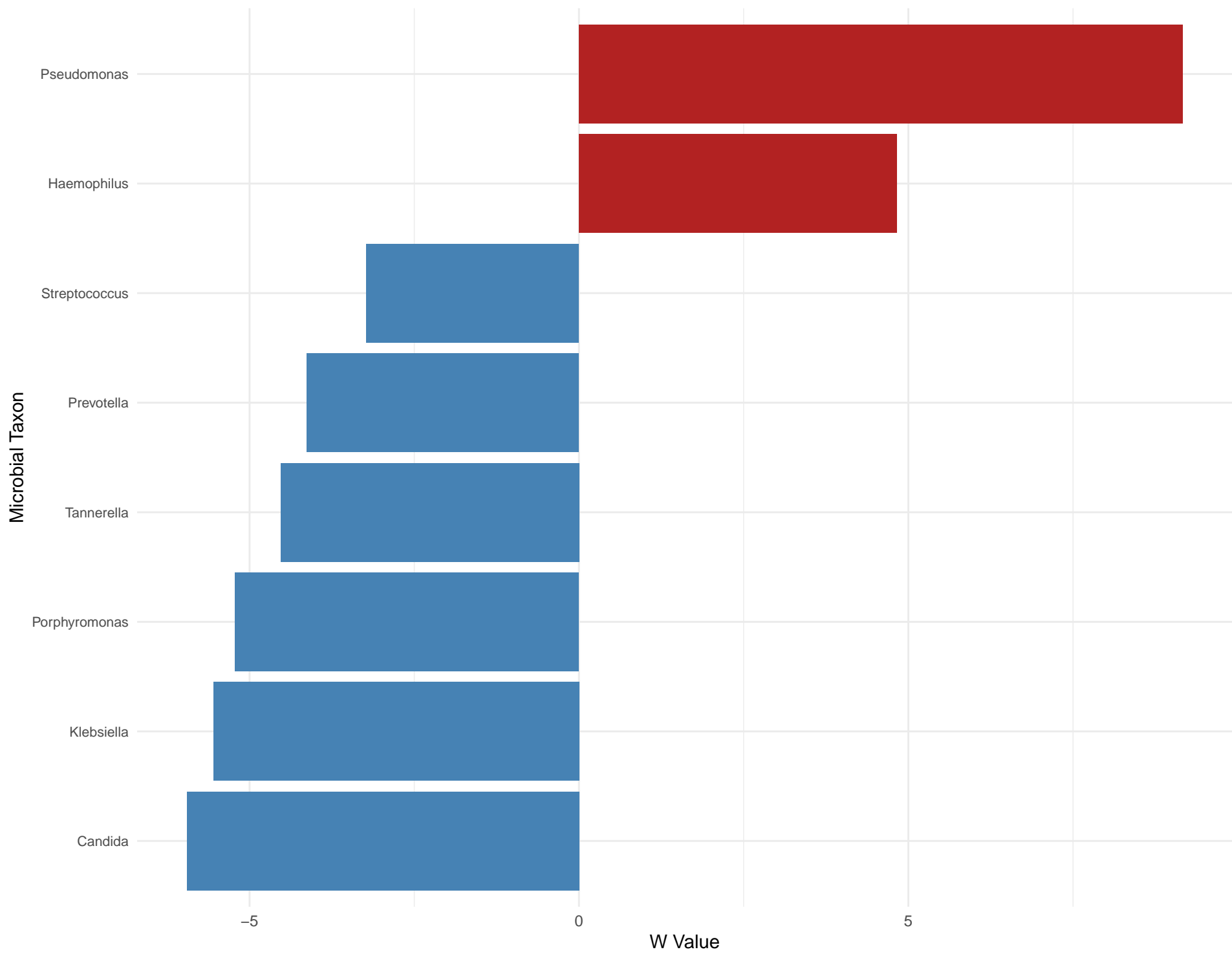

Supplement: Supplementary file 5 [file Data_Sheet_3.zip › ANCOMBC/ANCOMBC_W_value_plot.pdf]

A

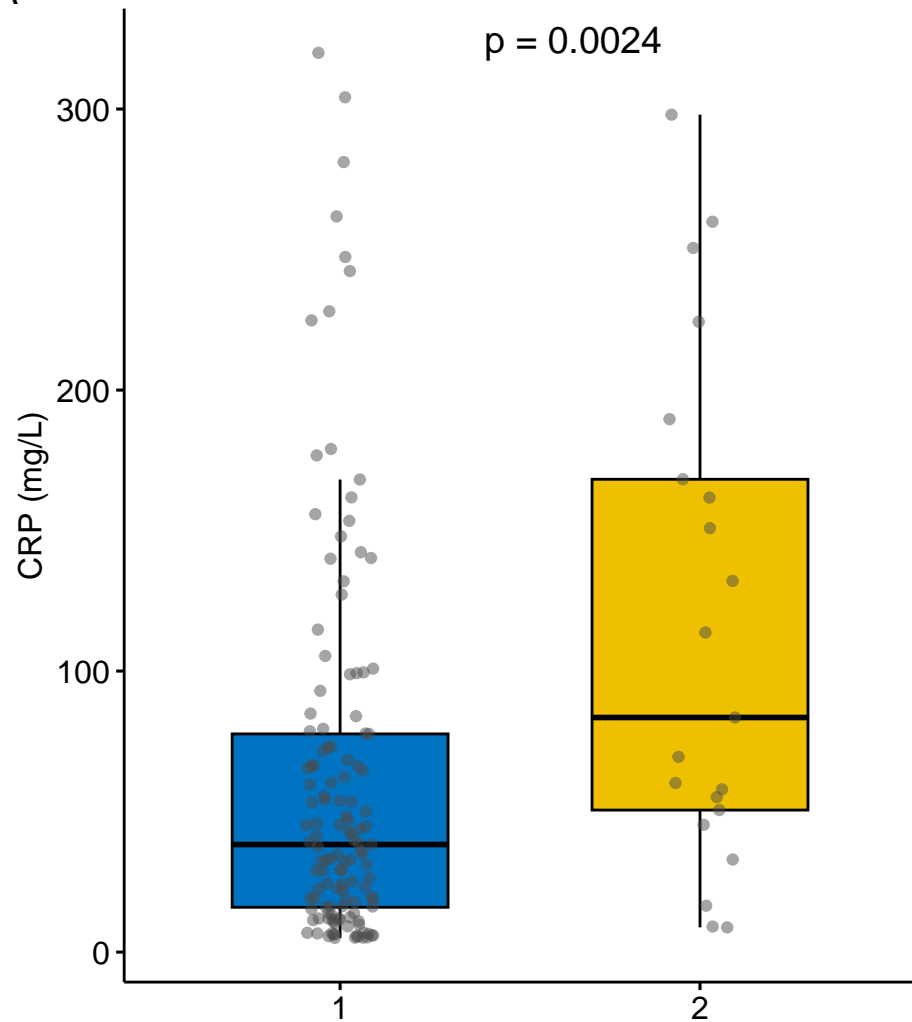

B

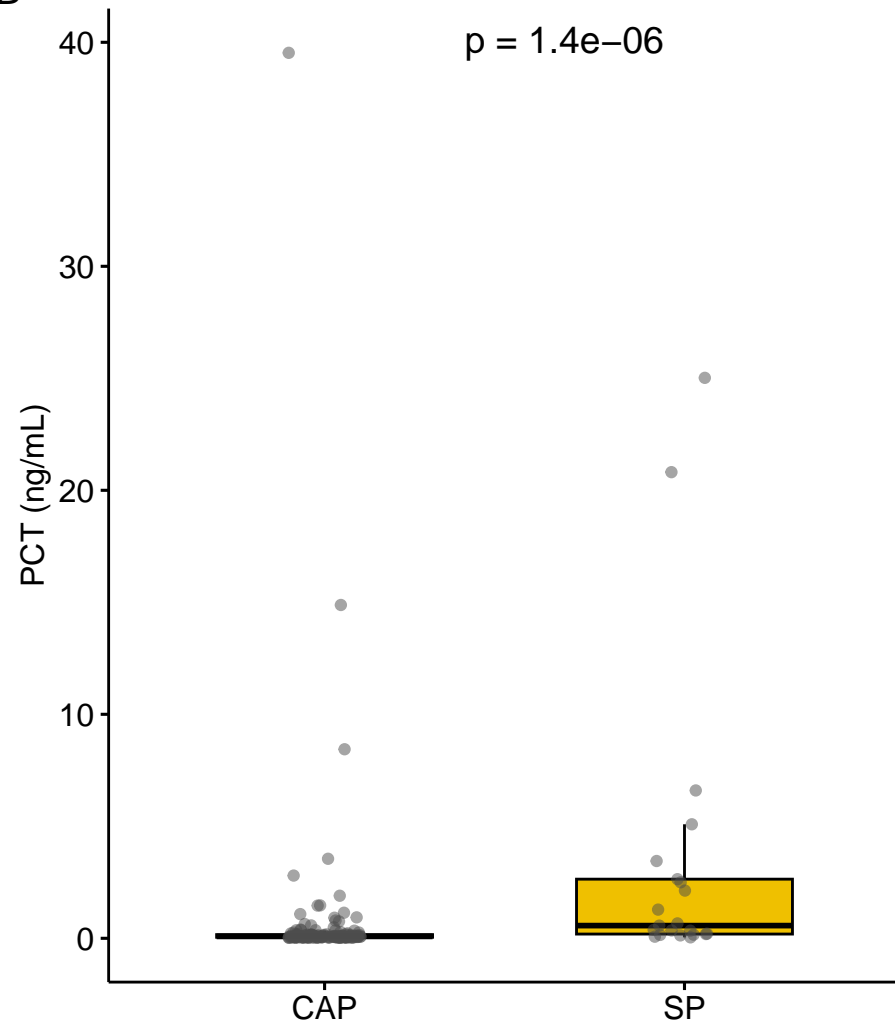

Supplement: Supplementary file 6 [file Data_Sheet_4.zip › corr/inflammatory_markers.pdf]

# Mantel Test: Correlation between CRP Differences and Microbial Dissimilarity

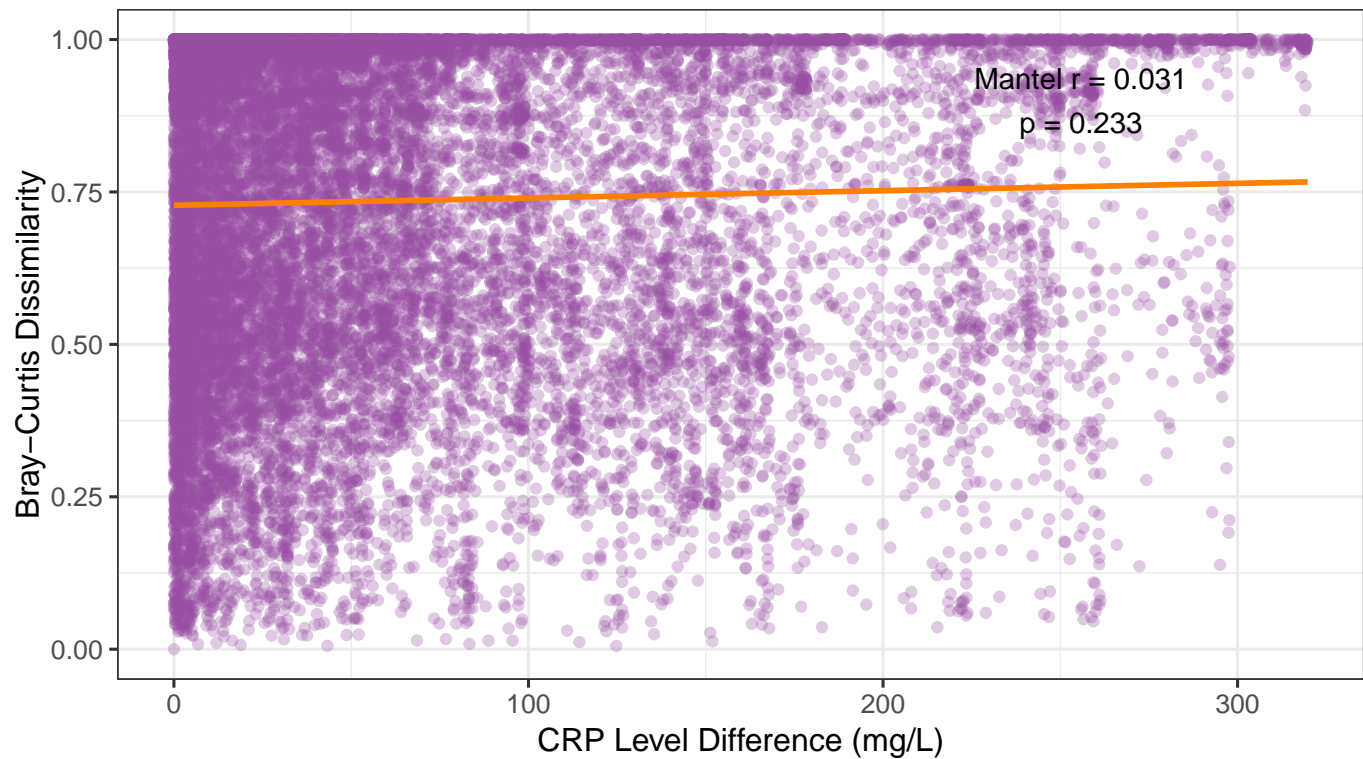

Supplement: Supplementary file 6 [file Data_Sheet_4.zip › corr/Mantel_Corr_CRP.pdf]

# Mantel Test: Correlation between PCT Differences and Microbial Dissimilarity

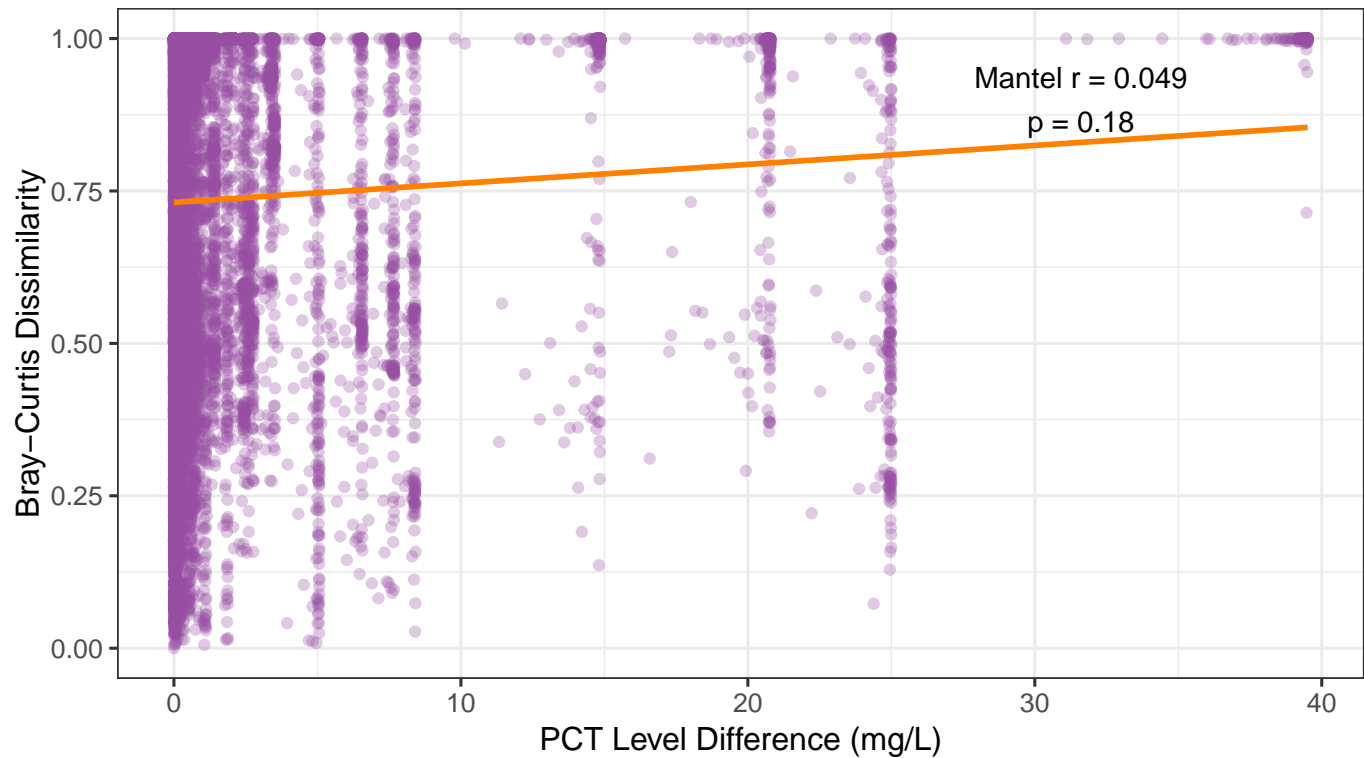

Supplement: Supplementary file 6 [file Data_Sheet_4.zip › corr/Mantel_PCT_Corr.pdf]

Study Group

CAP

SP

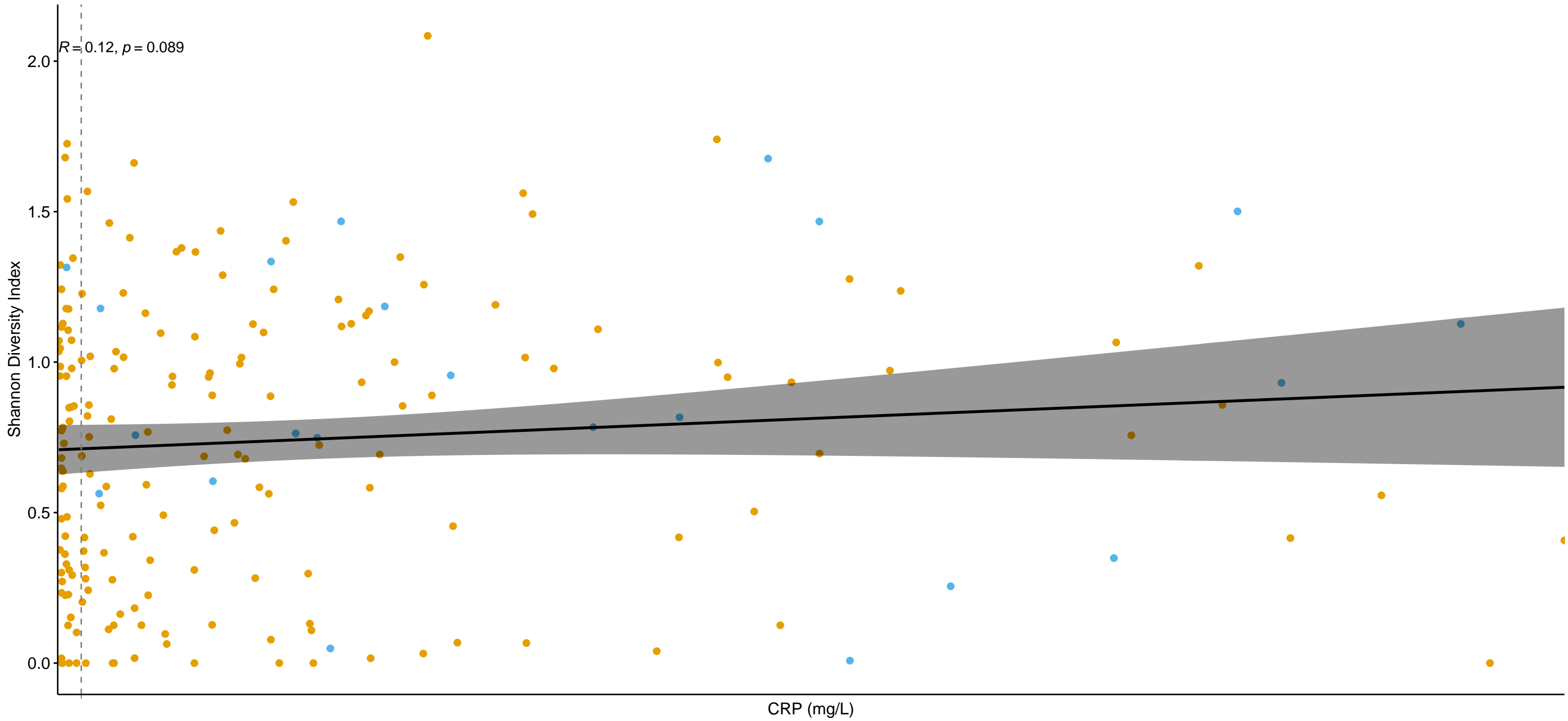

Supplement: Supplementary file 6 [file Data_Sheet_4.zip › corr/shannon_corr_CRP.plot.pdf]

Study Group

CAP

SP

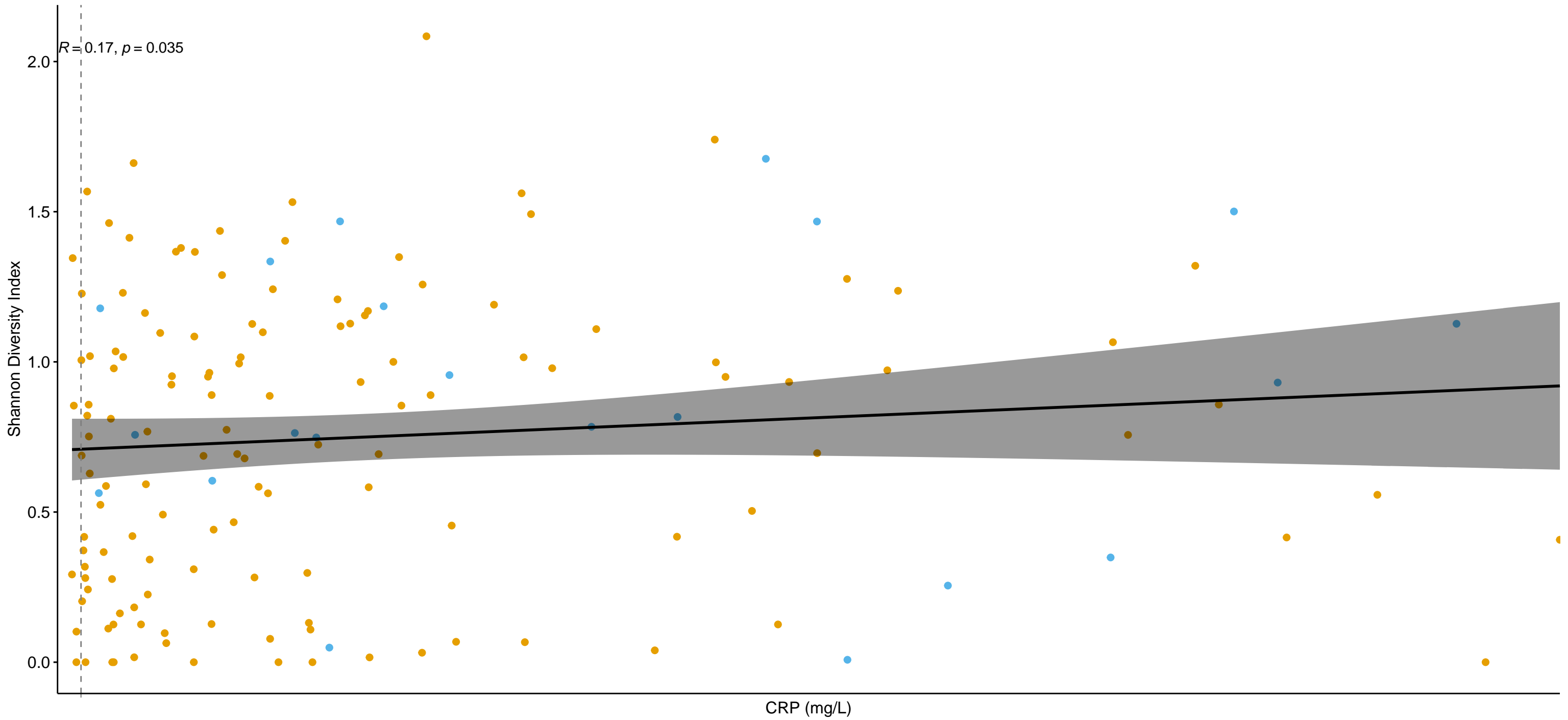

Supplement: Supplementary file 6 [file Data_Sheet_4.zip › corr/shannon_corr_CRP_cut3.plot.pdf]

Study Group

CAP

SP

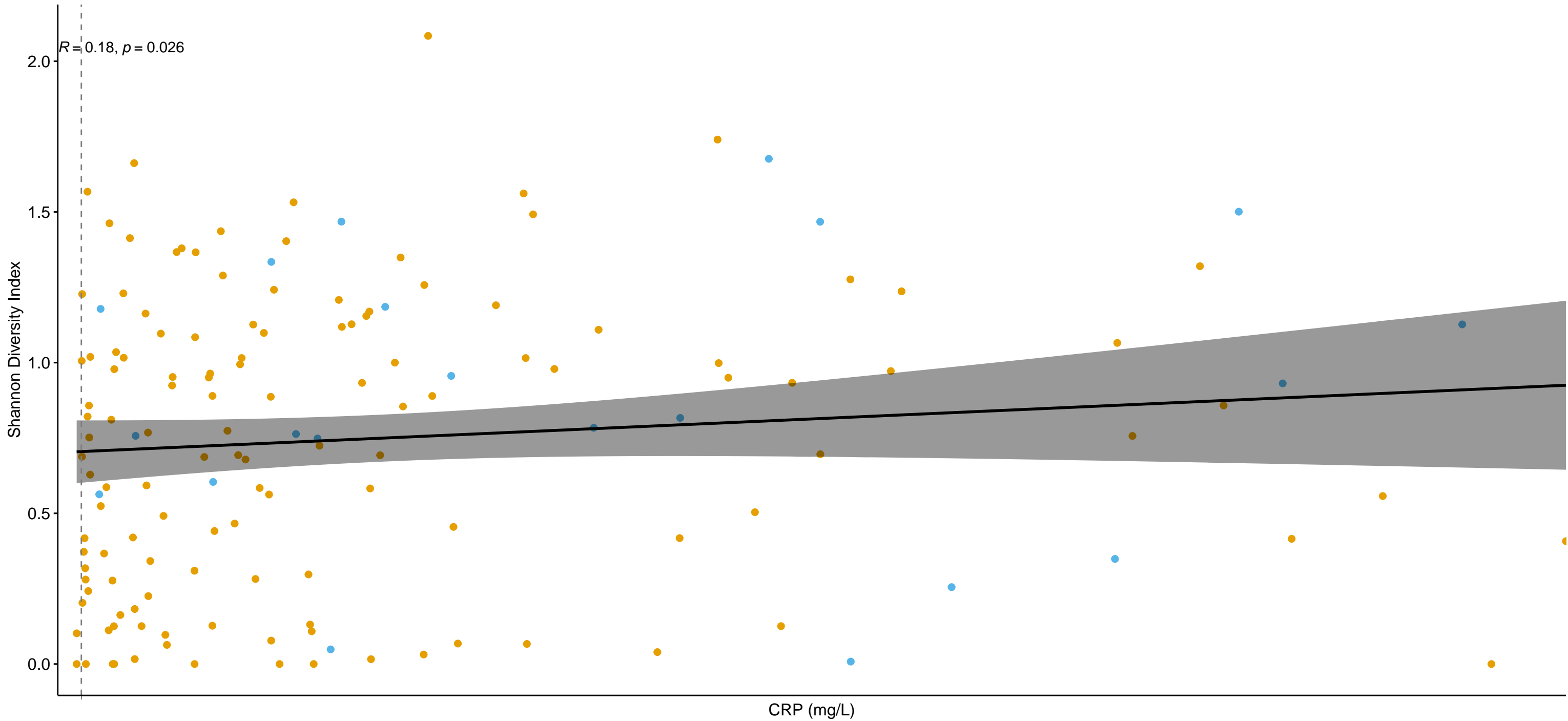

Supplement: Supplementary file 6 [file Data_Sheet_4.zip › corr/shannon_corr_CRP_cut4.plot.pdf]

Study Group

CAP

SP

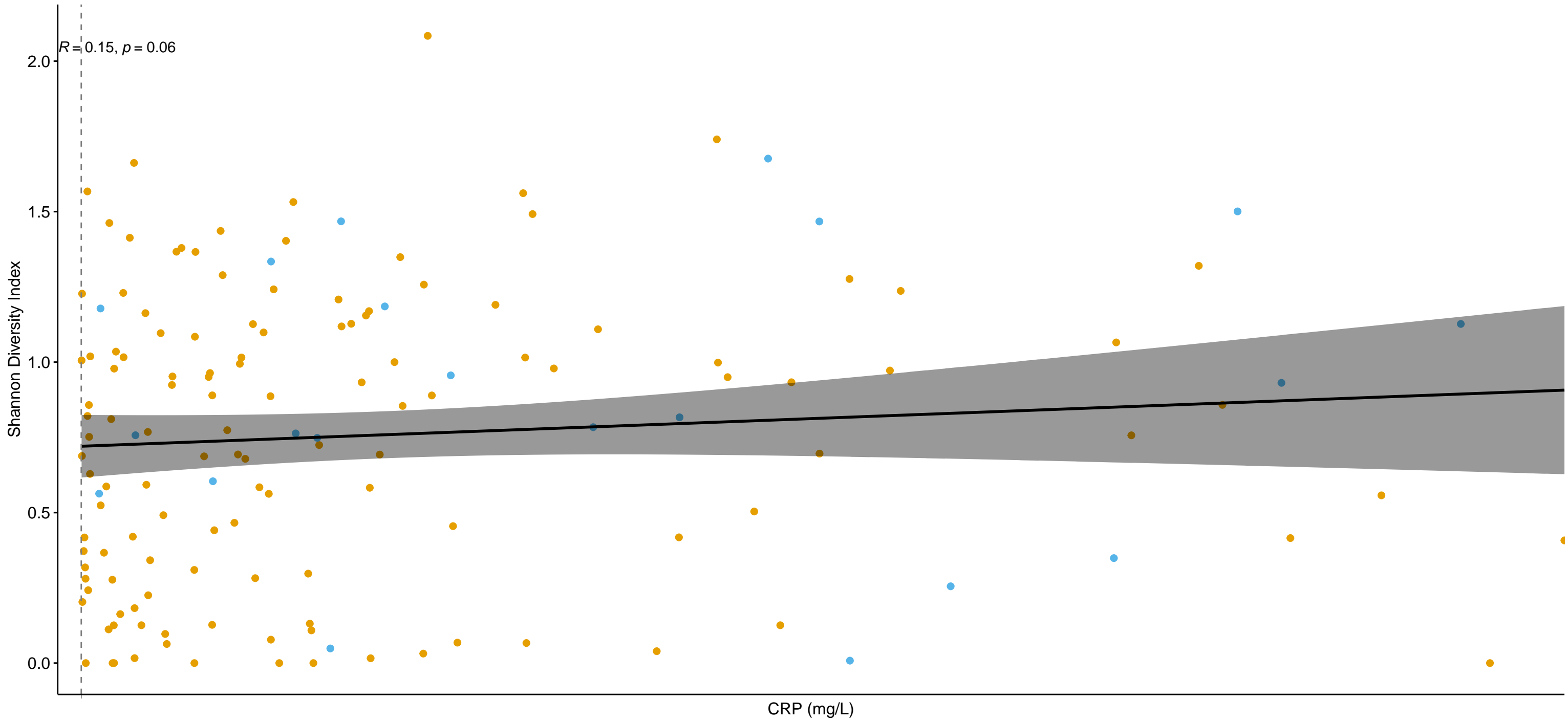

Supplement: Supplementary file 6 [file Data_Sheet_4.zip › corr/shannon_corr_CRP_cut5.plot.pdf]

Study Group   ● CAP   ● SP

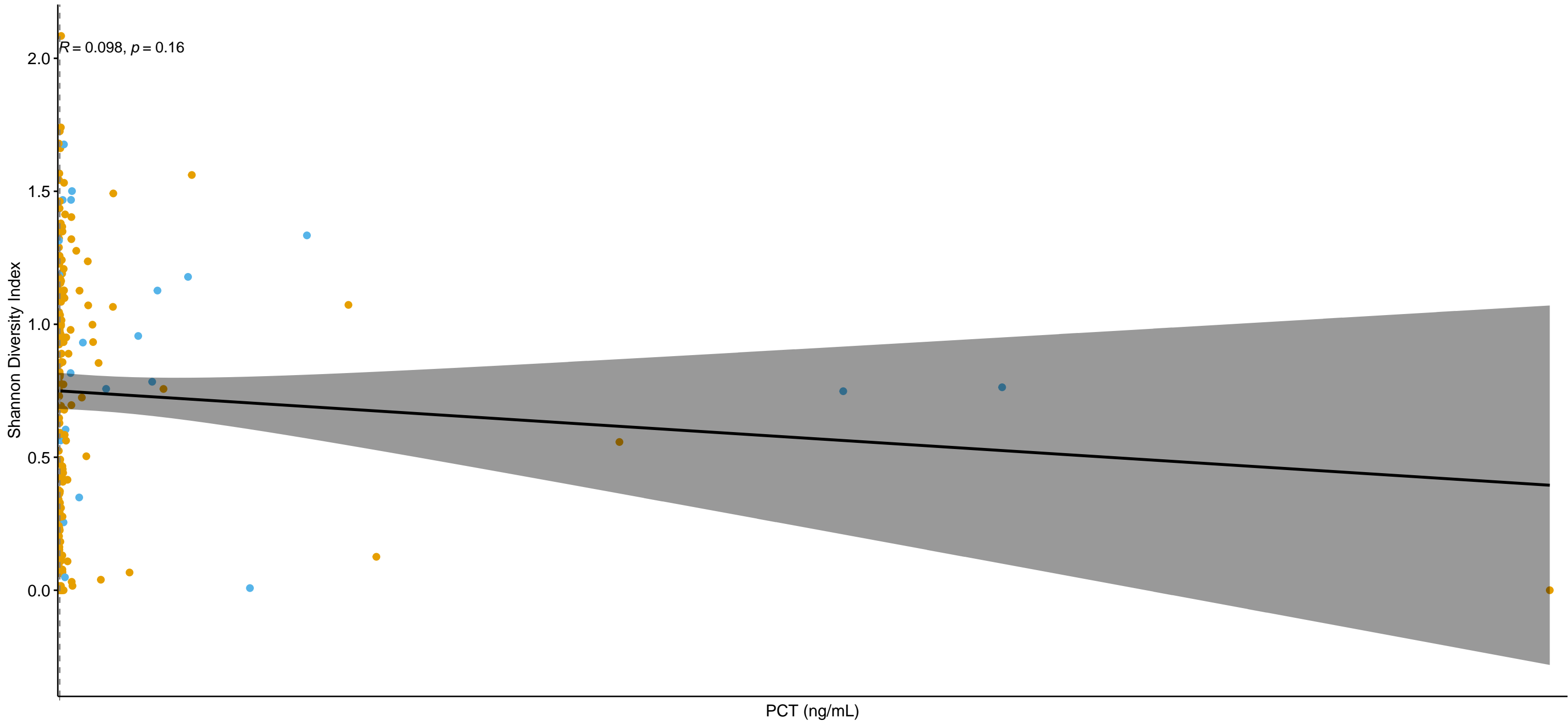

Supplement: Supplementary file 6 [file Data_Sheet_4.zip › corr/shannon_corr_PCT.plot.pdf]

Study Group    ● CAP    ● SP

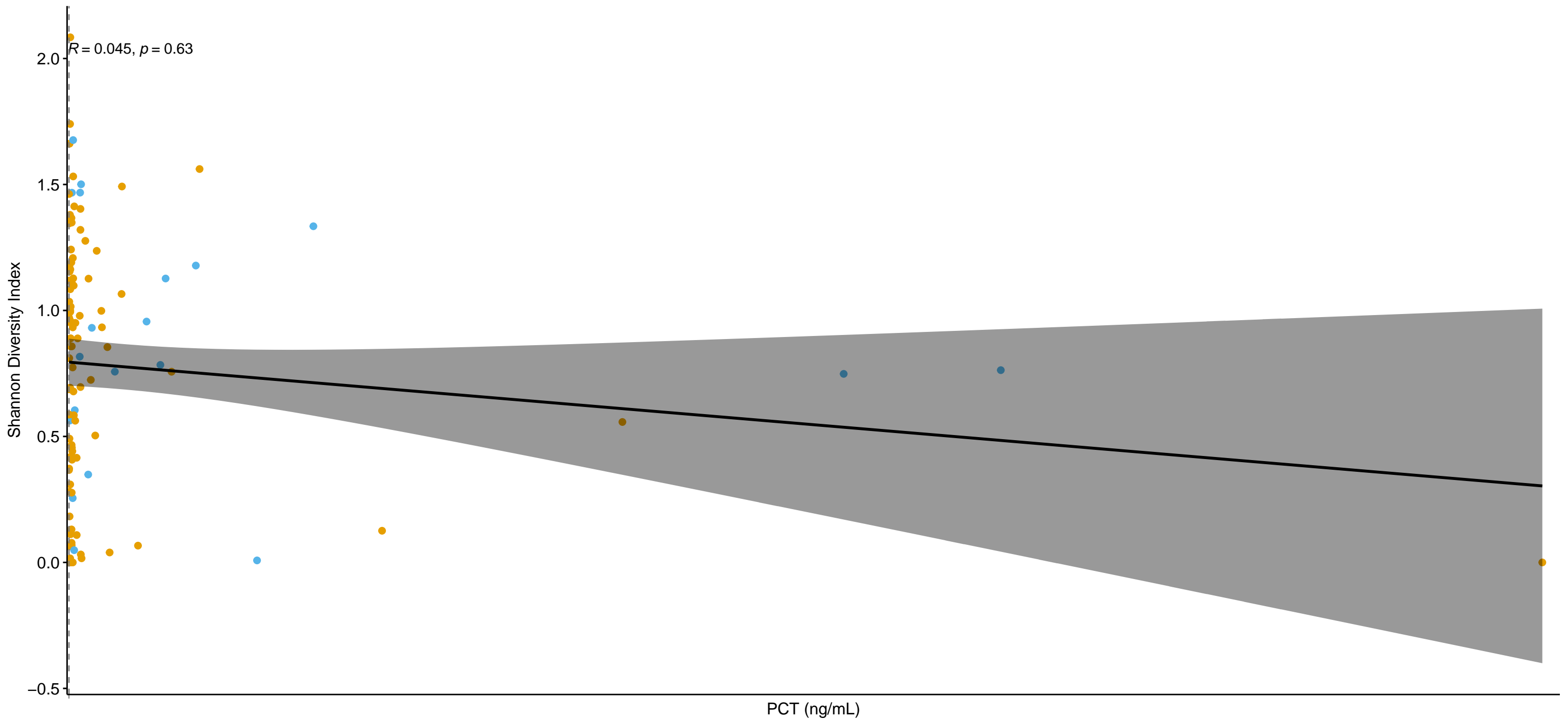

Supplement: Supplementary file 6 [file Data_Sheet_4.zip › corr/shannon_corr_PCT_cut0.05.plot.pdf]
