# Supplementary material for: Microbial and clinical disparities in pneumonia: insights from metagenomic next-generation sequencing in patients with community-acquired and severe pneumonia
Source: Front Microbiol. 2025 Jun 20;16:1538109. doi: 10.3389/fmicb.2025.1538109 (PMC12227010; doi:10.3389/fmicb.2025.1538109)

**Supplementary tables**

**Supplementary table 1 The sensitivity of mNGS compared to culture and specificity compared to clinical diagnosis**

|  | **Sensitivity compared to culture^a^** | | **Specificity vs. Clinical Non-Infectious Diagnosis^b^** |
| --- | --- | --- | --- |
|  | **CAP（N=204）** | **SP（N=25）** |  |
| mNGS | 91.3% (21/23) | 40% (2/5) | 0（0/1） |

^a^ The sensitivity of mNGS in comparison to traditional culture methods can be quantified as the ratio of mNGS-positive cases to culture-positive cases.

^b^ The specificity of mNGS in comparison to clinical diagnosis is defined as the proportion of mNGS-negative results among cases clinically diagnosed as non-infectious.

**Figure S1. Analysis of microbiome diversity and composition in CAP and SP patients.** (A-B) Box plot showing the distribution of C-reactive protein (CRP) levels and procalcitonin (PCT) levels in CAP and SP patients. (C-D) The Mantel test was used to assess the correlation between inflammatory differences and the microbial distance matrix, and no significant correlation was found.


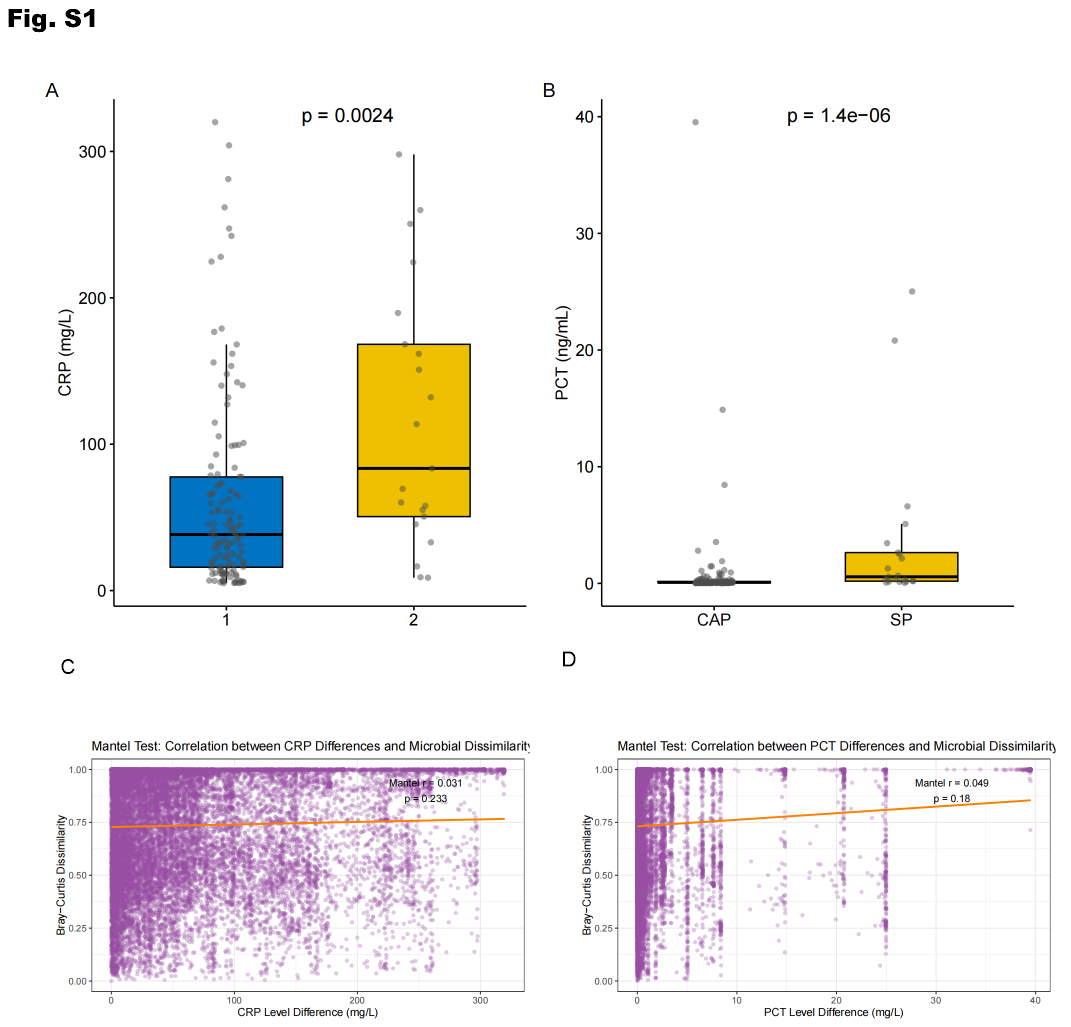

Supplement: Supplementary file 7 [file Data_Sheet_5.docx]
